# Investigating Possible Infectious Causes of Chronic Kidney Disease of Unknown Etiology in a Nicaraguan Mining Community

**DOI:** 10.4269/ajtmh.18-0856

**Published:** 2019-07-15

**Authors:** W. Katherine Yih, Martin Kulldorff, David J. Friedman, Jessica H. Leibler, Juan José Amador, Damaris López-Pilarte, Renee L. Galloway, Oriana Ramírez-Rubio, Alejandro Riefkohl, Daniel R. Brooks

**Affiliations:** 1 Department of Population Medicine, Harvard Medical School and Harvard Pilgrim Health Care Institute, Boston, Massachusetts;; 2 Division of Pharmacoepidemiology and Pharmacoeconomics, Department of Medicine, Harvard Medical School and Brigham and Women’s Hospital, Boston, Massachusetts;; 3 Division of Nephrology, Beth Israel Deaconess Medical Center, Harvard Medical School, Boston, Massachusetts;; 4 Department of Environmental Health, Boston University School of Public Health, Boston, Massachusetts;; 5 Department of Epidemiology, Boston University School of Public Health, Boston, Massachusetts;; 6 Centers for Disease Control and Prevention, Atlanta, Georgia

## Abstract

A chronic kidney disease of unknown etiology (CKDu) has been killing workers in Central America. Occupational heat stress is thought to play an important role. Leptospirosis and hantavirus have been suggested as additional possible risk factors. In a case–control study in a Nicaraguan mining community, a structured survey was administered to adults, and biological measurements and specimens were taken. Serum was analyzed for antibodies to *Leptospira* and hantavirus. Before statistical analysis, a board-certified nephrologist determined final case and control status based on serum creatinine and other laboratory values. Multivariable analysis was by logistic regression. In sensitivity analyses, cases were restricted to those diagnosed with CKDu in the previous 3 years. Of 320 eligible participants, 112 were classified as presumptive cases, 176 as controls and 32 as indeterminant. The risk of CKDu in those ever having worked in mining or construction was 4.4 times higher than in other participants (odds ratio = 4.44, 95% CI: 1.96–10.0, *P* = 0.0003). Eighty-three (26%) of the 320 participants were seropositive for at least one tested strain of *Leptospira*. No evidence of a causal link between leptospirosis or hantavirus and CKDu was found. The sensitivity analyses provide some evidence against the hypotheses that leptospirosis or hantavirus leads to CKDu *within a few years*. A major limitation was the impossibility of determining the absolute or relative timing of infection and CKDu onset. A prospective cohort design, with repeated collection of specimens over several years, could yield clearer answers about infections as potential etiologic agents in CKDu.

## INTRODUCTION

Since the mid-1990s or earlier, an epidemic of a chronic kidney disease of unknown etiology (CKDu) known as Mesoamerican nephropathy (MeN) has arisen in Pacific lowland areas from southern Mexico to Panama. Physical laborers such as sugarcane workers and miners, predominantly young and male, have been most affected.^1^ The same or a similar disease has been noted among agricultural workers in Sri Lanka^2,3^; Andhra Pradesh, India^4,5^; and Egypt.^6^ The risk profile of this disease of young workers, thus, differs markedly from that of the well-characterized chronic kidney disease (CKD) associated with diabetes, obesity, and older age. The disease is characterized by minimal proteinuria and biopsy findings that show primarily tubulointerstitial injury (sometimes with associated glomerular abnormalities). There is evidence to suggest that acute kidney injury (AKI) is also common in these workers, and repeated episodes of AKI may be an important factor in causing chronic kidney injury.^7^ The progression of disease is variable. Some cases originally diagnosed as MeN have shown improvement in kidney function, likely representing slowly resolving AKI. In some cases, kidney function remains impaired but stable for years, whereas in others, it progresses rapidly to end-stage renal disease.^7,8^ It is difficult for families to even maintain adequate palliative care in resource-poor settings.^9^ The present epidemic in the Americas is estimated to have killed more than 20,000 people^10^ and has deprived families of husbands, fathers, income, and savings, decimating whole communities.

There is broad consensus among researchers that the etiology of MeN has an occupational component, with heat stress likely playing an important role.^11,12^ However, it is generally accepted that other risk factors might also be involved because MeN appears to be of relatively recent origin and geographically restricted, whereas exposure to occupational heat stress is not new and is widely distributed around the globe.

It has been hypothesized that an infectious disease may play a role in the development of MeN and similar epidemics of CKDu.^13–18^ Leptospirosis has been a prime suspect, owing to the frequent involvement of the kidney and the prevalence of leptospirosis in the kinds of occupations that have been implicated in MeN. Leptospirosis is a bacterial zoonotic disease caused by pathogenic species of the spirochete *Leptospira*, of which there are more than 250 pathogenic serovars.^19^ It is one of the leading zoonotic causes of morbidity and mortality in humans worldwide, with more than one million cases and close to 60,000 deaths annually.^20^ Leptospirosis is most prevalent in agricultural areas and poor urban areas of the tropics, related to the high density of animal reservoirs and inadequate sanitation. Many species of mammals carry and excrete the bacteria in urine; rodents are recognized as a major source of infection to humans.^21^ The bacteria enter new hosts, including humans, via the skin and mucous membranes. Occupational risk groups include agricultural workers, animal husbandry workers, fish workers, miners, and sewer workers,^22,23^ and the bulk of the global burden of disease is borne by males aged 20–49 years.^20^ Flooding can lead to outbreaks. Over the past 20 years, multiple outbreaks of leptospirosis have occurred in areas where MeN is common, and leptospirosis is now endemic in at least one such area, the Department of León in Nicaragua.^24^

Presentations of leptospirosis in humans range from asymptomatic to fulminant fatal disease. Cases are often misdiagnosed because of the nonspecific nature of the symptoms.^22^ Renal involvement is typical and is commonly characterized by tubulointerstitial nephritis and tubular dysfunction.^25–27^ The bacteria enter the kidney, ultimately penetrating the renal tubules to adhere to their inner surface. Markers of kidney function such as serum creatinine typically return to normal levels during the second week of illness, as the infection is cleared.^28–30^ However, renal function may continue to deteriorate after AKI, at least in the presence of other contributing factors,^31–33^ and there is evidence that AKI—in spite of early normalization of serum creatinine—can increase the risk of developing CKD.^34–36^ Sequelae of AKI specifically from leptospirosis are known to occur,^27,37^ and a recent study claims that chronic or repeated leptospirosis infection is associated with both prevalence and severity of CKD.^36^

Thus, the hypothesis that leptospirosis is a risk factor for developing MeN is biologically plausible and its credibility bolstered by similarities in the demographic, geographic, and temporal distributions of the two diseases.

Hantavirus is another rodent-borne pathogen that has been proposed as a possible causal agent in CKDu.^15^ Old World varieties of hantavirus have tended to be associated with hemorrhagic fever with renal syndrome; the Old World Puumala hantavirus is well-known to induce an acute tubulointerstitial nephritis and AKI.^38^ In Sri Lanka, using serology to determine past hantavirus exposure in a descriptive cross-sectional study, Gamage et al.^39^ found that past exposure to hantavirus was significantly higher among patients with CKDu than among a control group. In contrast to Old World varieties, New World varieties of hantavirus have tended to be associated with cardiopulmonary syndrome; however, Old World hantaviruses have been reported in the Americas, and novel strains of hantavirus continue to be identified.^15^

The main objective of our study was to test the association between leptospirosis and evidence of reduced renal function in a region of Nicaragua heavily affected by MeN, but we also investigated hantavirus exposure, self-reported occupational exposures, and other potential risk factors.

## MATERIALS AND METHODS

We conducted a case–control study among adult residents of a gold-mining area comprising three towns and adjoining rural zones and an approximate population of 10,000 in the Department of León, Nicaragua. We sought to enroll at least 300 subjects, including 100–150 MeN cases, aged 18 years or older and without diabetes or other conditions known to cause CKD. Formal random selection of cases and controls was not feasible in this setting. The initial, field-based determination of case and control status was made on the basis of records held by the local government health center or self-report during the field team’s recruitment efforts. To identify potential cases, we relied primarily on referral by the health center and secondarily on community referral. To identify potential controls, we used primarily community referral, often receiving suggestions from cases’ family members of relatives or neighbors to recruit as controls. We started by enrolling presumptive cases and then, after several dozen presumptive cases had been enrolled, we began to enroll controls as well, checking the demographics of both groups every few days to ensure similar age and gender distributions in the controls as in the cases. (In operational terms, we sorted our spreadsheet of participants, counted up the cases in each gender and age group, and calculated proportions in each group. We then did the same for controls. We then compared the age and gender distributions in cases and controls. If, for example, we saw that 17% of the cases and 33% of the controls were in their 30s and 35% of the cases but only 12% of the controls were in their 50s, we targeted older people in our recruitment of controls over the subsequent few days.) Informed consent was obtained from all participants.

The field team measured weight and blood pressure and collected an 8-mL tube of venous blood and a sample of urine. The blood was centrifuged, and the serum was separated into aliquots in 2-mL cryovials, frozen, and kept at −20°C for 2–3 days at the local health center. The serum specimens were then transported on icepacks to the Centro Nacional de Diagnóstico y Referencia (CNDR) of the Nicaraguan Ministry of Health in Managua, where they were maintained at −80°C until further processing. One of the cryovials was used to measure serum creatinine, creatine phosphokinase, glucose, and uric acid at the CNDR. Another cryovial was sent on dry ice to the CDC in Atlanta. At CDC, a laboratory technician performed the microscopic agglutination test (MAT), the standard reference test for serological diagnosis of leptospirosis,^40^ assessing titers of antibodies to the 20 strains in the CDC’s standard panel and to four additional strains included at our request. The MAT entails incubating live leptospiral cell suspensions with serially diluted serum specimens and reading the resulting agglutination titers using dark-field microscopy. The highest dilution of serum that agglutinates ≥ 50% of the cells for each strain tested is the titer reported for that strain.^41^ We considered all *Leptospira* antibody titers of ≥ 1:100 to be positive, representing evidence of past exposure, as is conventional.^36,40^ The remainder of the serum sample sent to CDC was used for hantavirus serology.^42^ All samples were run twice for Sin Nombre IgG, which has broad cross-reactivity, including with Old World hantavirus strains, and once for Laguna Negra IgG. To be considered positive for hantavirus, a sample had to meet two criteria: a titer of ≥ 1:400 and a sum optical density of the 1:100, 1:400, 1:1,600, and 1:6,400 titers of > 0.95.

The urine specimen was analyzed on-site using dipstick measurements of glucose, red and white blood cells, nitrites, and protein. Before dipstick measurements, ∼2-mL and ∼10-mL aliquots were poured into a 2-mL cryovial and a 15-mL conical tube, respectively, frozen, and kept at −20°C for 2–3 days at the local health center, and then transported on icepacks to the CNDR and maintained there at −80°C. For individuals with trace or greater proteinuria by dipstick, the ∼2-mL aliquot was used to measure protein and creatinine at the CNDR. The ∼10-mL aliquot of urine (a convenience sample of 288) was sent to the CDC on dry ice and used for *Leptospira* polymerase chain reaction (PCR) testing. We considered it worthwhile to conduct PCR to complement the MAT, in view of the fact that at least two studies had presented evidence of urinary shedding of *Leptospira* by asymptomatic, MAT-negative individuals, using PCR to identify leptospiral DNA in urine samples.^43,44^ Analyses were conducted using the method, including the primer/probe sequences, described by Stoddard et al.^45^ and modified to increase sensitivity as described by Galloway and Hoffmaster.^46^

The clinicians on the field team returned the results of the serum and urine tests conducted in Nicaragua to the participants, interpreting them and recommending medical follow-up as appropriate.

At the time of biological sample collection, the field team administered a survey to study participants, collecting demographic information, residential and occupational histories, family history, exposure to animals, use of nonsteroidal anti-inflammatory drugs, smoking, and other possibly relevant exposures. In assembling the dataset, we grouped occupations into mining, masonry/construction, work with animals, small-scale agriculture, large-scale (plantation) agriculture, and “others.” “Others” consisted of diverse occupations that we deemed to involve less physical exertion, heat stress, and dehydration than the more specific job categories, such as driver, security guard, mechanic, and domestic worker. All other variables explored in the statistical analysis were either continuous or dichotomous (e.g., yes/no).

After the completion of field-work but before determination of leptospirosis and hantavirus exposure status and any statistical analysis, one of the authors (D. J. F.), a board-certified nephrologist with experience working in this population, made the final designations of presumptive MeN case, control, and borderline or indeterminate based on information and measurements conducted on serum and urine samples obtained on the day of recruitment. Measurements taken into consideration were serum creatinine, estimated glomerular filtration rate (eGFR) based on the Chronic Kidney Disease Epidemiology Collaboration equation,^47^ serum glucose (non-fasting), proteinuria and hematuria by urine dipstick, and urine protein/creatinine ratio, as well as the subject’s weight, age, and self-reported previous creatinine values. Participants with diabetes or other condition known to cause CKD were excluded. Those with borderline measurements were excluded from all statistical analyses other than a descriptive analysis of leptospirosis seropositivity. With few exceptions, these clinically informed determinations agreed with the simpler, purely creatinine-based criteria of 1) current creatinine ≤ 1.1 mg/dL = control, 2) current creatinine > 1.1 and < 1.5 = excluded from main analysis, and 3) current creatinine ≥ 1.5 mg/dL = case (Table 1**)**. The exceptions included instances of borderline creatinine values where extremes in age led to a discordance between case/control status and eGFR, extremes of weight that increased the likelihood of misclassification due to uncertain muscle mass, or abnormal urine findings in people who otherwise could have been considered controls. We acknowledge that in most instances, the final determination of MeN status was made on the basis of only one (the current) serum creatinine value; we lacked the resources to determine whether kidney function (as measured by serum creatinine and eGFR) was impaired for ≥ 3 months, a requirement for meeting the case definition of the Pan American Health Organization.^48^

**Table 1:** Comparison of what Mesoamerican nephropathy (MeN) classifications of study participants would have been if purely creatinine-based criteria had been used (top row) and final classifications made by nephrologist using additional measurements and clinical judgment (bottom row), showing additions and subtractions

	Controls	Borderline, excluded	Cases	Creatinine values of participants whose categories changed (mg/dL)
Using current creatinine value*	178	29	113	–
Changed from control to excluded	−2	+2	N.a.	1.1
Changed from excluded to case	N.a.	−2	+2	1.3 (in women)
Changed from case to excluded	N.a.	+3	−3	1.5 or 1.6
Final†	176	32	112	–

* Current creatinine ≤ 1.1 mg/dL = control, current creatinine > 1.1 and < 1.5 = excluded from main analysis, and current creatinine ≥ 1.5 mg/dL = case.

† A board-certified nephrologist with experience working in this population (D. J. F.) made the final designations of presumptive MeN case, control, and borderline/to-be-excluded based on information and measurements conducted on serum and urine samples obtained on the day of recruitment.

Our main statistical analyses, incorporating only cases and controls, were by logistic regression, with (presumptive) MeN (yes/no) as the dependent variable. We included seropositivity for any of the 24 tested *Leptospira* strains as one of the independent variables. The other independent variables tested in model-building came from the survey and were added one by one to a basic model that included age and gender, to assess their impact on MeN status. Plausible interactions were also checked one by one. Model fit was assessed throughout, using the Akaike information criterion. The covariates/interaction terms found to be influential regarding MeN status were retained in the final model. In view of the issue of waning antibodies to *Leptospira*, we did a post hoc set of analyses, restricting to the cases with a MeN diagnosis in the previous 3 years and including all the controls. Hantavirus seropositivity was studied only descriptively and was not included in the regression analyses.

The study was approved by the Institutional Review Boards of Boston University Medical Center and the Nicaraguan Ministry of Health.

## RESULTS

### Demographics of cases and controls.

We enrolled 322 people in the study. Two were later determined to be ineligible because of high blood sugar, leaving 320 participants, of whom 297 were male and 23 were female. Of the 320, the nephrologist in our team (D. J. F.) classified 112 as cases and 176 as controls. The other 32 participants had biological measurements that made their case/control status uncertain and were excluded from the main analyses. Males comprised 94% of cases and 91% of controls. The median age was 48.5 years (range: 21–65) in cases and 42.5 (range: 19–76) in controls. Although there were differences in the age distributions (Table 2), the overall age range in each of the groups was sufficiently broad as to allow for age adjustment in the regression analyses.

**Table 2:** Characterization of study population by gender, age, and presumptive Mesoamerican nephropathy status

	Cases		Controls		Indeterminates		Total	
	Number	Proportion (%)	Number	Proportion (%)	Number	Proportion (%)	Number	Proportion (%)
Gender								
Male	105	93.8	160	90.9	32	100.0	297	92.8
Female	7	6.2	16	9.1	0	0.0	23	7.2
Age group								
18–29 years	4	3.6	28	15.9	4	12.5	36	11.3
30–39 years	22	19.6	46	26.1	5	15.6	73	22.8
40–49 years	34	30.4	44	25.0	10	31.3	88	27.5
50–59 years	37	33.0	39	22.2	8	25.0	84	26.3
60–69 years	15	13.4	17	9.7	5	15.6	37	11.6
≥ 70 years	0	0.0	2	1.1	0	0.0	2	0.6
Total	112	100	176	100	32	100	320	100

### *Leptospira* serology and PCR.

**Figure 1.**
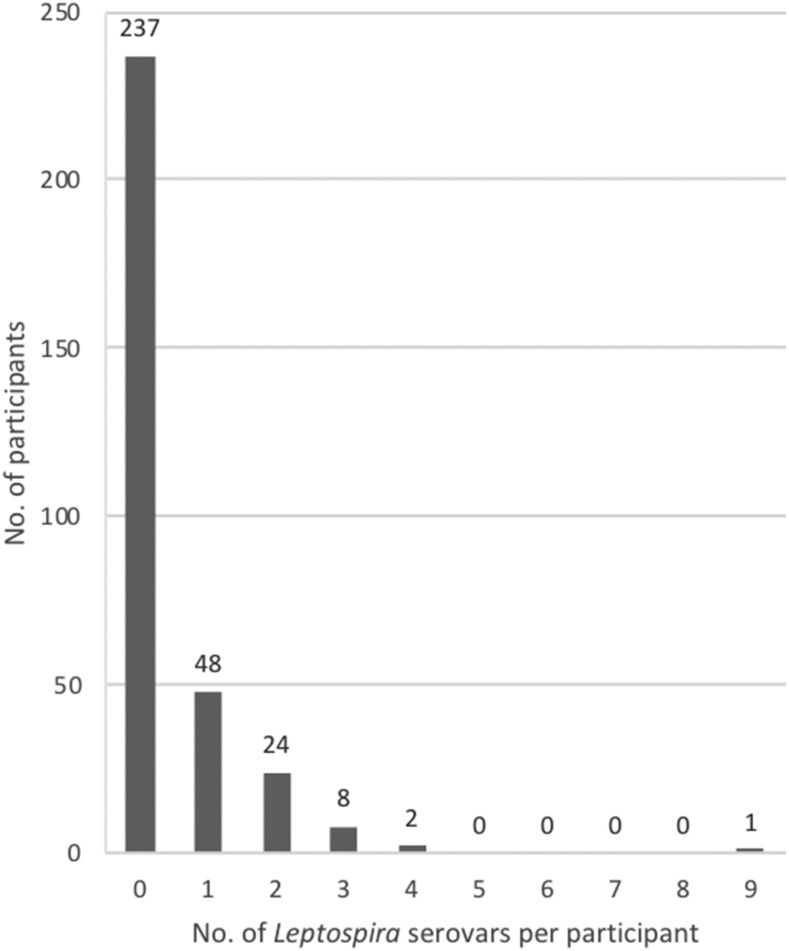
Number of participants seropositive for *Leptospira* by number of serovars for which they were seropositive (*n* = 320).

Of the 7,680 MATs performed (320 participants × 24 *Leptospira* strains), 137 were positive. Eighty-three (26%) of the 320 participants were seropositive for at least one strain. Seropositivity to any strain, by participants’ demographic and occupational characteristics and MeN status, is shown in Table 3. The number of strains for which a participant was seropositive ranged from 0 (for 237 participants) to 9 (for one participant) (Figure 1). The strain for which the most participants were positive was *Leptospira noguchii* serovar Nicaragua strain 1011 (*n* = 57; 18% of participants). The next most frequent strain was *Leptospira interrogans* serovar Bratislava strain Jez-Bratislava, with 11 seropositive participants (Table 4). Of the 112 MeN cases, 27 (24%) were seropositive for any of the 24 *Leptospira* strains for which testing was performed; of the 50 MeN cases diagnosed within the previous 3 years, 15 (30%) were seropositive; and of the 176 controls, 54 (31%) were seropositive (Table 3). All urine specimens analyzed for leptospiral DNA by PCR were negative, indicating absence of acute infection in this population.

**Table 3:** Seropositivity to any strain of *Leptospira*, by demographic and occupational characteristics and MeN status

	Total	Number negative	Number positive	Proportion positive (%)
Gender				
Male	297	215	82	27.6
Female	23	22	1	4.3
Age group				
18–29 years	36	28	8	22.2
30–39 years	73	57	16	21.9
40–49 years	88	61	27	30.7
50–59 years	84	62	22	26.2
60–69 years	37	27	10	27.0
≥ 70 years	2	2	0	0.0
Occupation (ever—categories not mutually exclusive)				
Mining	191	148	43	22.5
Masonry	108	77	31	28.7
Livestock-tending	143	95	48	33.6
Agriculture	205	142	63	30.7
Other	115	90	25	21.7
MeN status				
Case	112	85	27	24.1
Case diagnosed within previous 3 years	50	35	15	30.0
Control	176	122	54	30.7
Indeterminate, excluded	32	30	2	6.2
Total participants	320	237	83	25.9

MeN = Mesoamerican nephropathy. Occupations refer to occupations ever engaged in and are not mutually exclusive.

**Table 4:** Number of study participants seropositive for each *Leptospira* strain in the MAT conducted at the CDC by MeN status (*n* = 320)

*Leptospira* species, serovar, and strain	No. of MAT-positive participants			
	Total	MeN cases	MeN controls	MeN indeterminate
CDC standard panel (20 strains):				
*L. borgpetersenii* serovar Ballum strain Mus 127	0	0	0	0
*L. borgpetersenii* serovar Javanica strain Veldrat Bataviae 46	0	0	0	0
*L. borgpetersenii* serovar Tarassovi strain Perepelitsin	6	3	3	0
*L. interrogans* serovar Australis strain Ballico	3	2	1	0
*L. interrogans* serovar Autumnalis strain Akiyami A	0	0	0	0
*L. interrogans* serovar Bataviae strain Van Tienen	5	1	3	1
*L. interrogans* serovar Bratislava strain Jez-Bratislava	11	5	6	0
*L. interrogans* serovar Canicola strain Ruebush	8	3	5	0
*L. interrogans* serovar Djasiman strain Djasiman	3	0	3	0
*L. interrogans* serovar Grippotyphosa (no strain name)	2	1	1	0
*L. interrogans* serovar Icterohaemorrhagiae strain RGA	1	0	1	0
*L. interrogans* serovar Mankarso strain Mankarso	8	1	7	0
*L. interrogans* serovar Pomona strain Pomona	4	3	1	0
*L. interrogans* serovar Pyrogenes strain Salinem	6	1	5	0
*L. interrogans* serovar Wolffi strain 3705	3	0	3	0
*L. kirschneri* serovar Cynopteri strain 3522 C	0	0	0	0
*L. santarosai* serovar Alexi strain HS 616	7	1	5	1
*L. santarosai* serovar Borincana strain HS 622	1	0	1	0
*L. santarosai* serovar Georgia strain LT 117	7	1	6	0
*L. weilii* serovar Celledoni strain Celledoni	0	0	0	0
Additional strains (4):				
*L. biflexa* serovar Patoc strain Patoc I	3	0	3	0
*L. interrogans* serovar Copenhageni strain M20	2	0	2	0
*L. noguchii* serovar Nicaragua strain 1011	57	20	36	1
*L. santarosai* serovar Shermani strain 1342K	0	0	0	0
Total positive MATs (in 83 of 320 participants)	137	42	92	3

MAT = microscopic agglutination test; MeN = Mesoamerican nephropathy. Totals at bottom represent positive MATs, not unique individuals.

### Statistical analysis.

In logistic regression modeling for MeN risk incorporating the 112 cases and 176 controls, gender was not statistically significant, but we retained it throughout. We did not find any statistically significant associations between any of the following self-reported conditions and MeN, and these were removed from subsequent modeling: smoking, dysuria, use of nonsteroidal anti-inflammatory drugs, or rats or mice in the household. Regarding occupations, ever having worked in mining conferred an approximately 2-fold increased risk of MeN compared with never-mining (odds ratio [OR] = 2.19, 95% CI: 1.09–4.39, *P* = 0.027). A similar risk was seen for ever having worked in construction compared with never-in-construction (OR = 2.05, 95% CI: 1.01–4.16, *P* = 0.046). When we combined mining and construction work into a single mining/construction variable, we found that the risk of MeN in those ever having worked in either industry was more than four times higher than in those never having worked in either (OR = 4.44, 95% CI: 1.96–10.0, *P* = 0.0003). Among miners/construction workers, there was an inverse relationship between the number of years worked in these industries and risk of MeN, with the risk decreasing by about 4.7% with every additional year worked (*P* = 0.0081). No significant difference in risk was observed for any of the other occupations, namely, work with animals, small or large-scale agriculture (which did not include sugarcane production for any of our participants), or the composite “other” job category.

*Leptospira* seropositivity was negatively associated with MeN (OR = 0.56, 95% CI: 0.31–1.01, *P* = 0.054) (Supplemental Table 1). In the final logistic regression model, which included age, age-squared, gender, mining/construction (yes/no), years worked in mining/construction, *Leptospira* seropositivity, and an interaction term between mining/construction (yes/no) and seropositivity, the negative association was more pronounced among those who had never worked in mining or construction (OR = 0.08, 95% CI: 0.01–0.68, *P* = 0.02) (Supplemental Table 2) than in ever miners/construction workers (OR = 0.77, 95% CI: 0.41–1.46, *P* = 0.43) (Supplemental Table 3). The results of the comparable set of analyses in which MeN cases were restricted to those diagnosed in the prior 3 years were qualitatively similar.

### Hantavirus serology.

We did not observe an association between hantavirus exposure and MeN. Overall, 42 (24%) of the 176 controls and 18 (16%) of the 112 MeN cases had a positive result in any of the three runs; almost all of these were positive in at least one of the Sin Nombre runs. Four participants were positive for the Laguna Negra strain (two of which were also positive for Sin Nombre in at least one of those two runs); they were all MeN controls. The CDC hantavirus serology experts noted that the positivity rate seemed quite high and that most of the positive samples had a titer of only 1:400 (the minimum required to qualify as “positive”), leading them to doubt that all the observed positives were true positives. They had observed turbidity in some specimens and speculated that fungal contamination and/or the bacterial coating on the plates could have caused some cross-reactivity and false positives.

## DISCUSSION

In this study of 112 MeN cases and 176 controls in a mining area of Nicaragua, we found a high rate of *Leptospira* seropositivity among study participants but did not find a positive association between *Leptospira* seropositivity and MeN. The data were consistent with a protective effect, driven mainly by a statistically significant negative association among those who had not worked in mining or construction. Because it is highly unlikely that *Leptospira* infection is protective against kidney disease, the negative association is likely due to chance or bias. Reverse causation is one possibility. For example, cases (perhaps especially those without a history of physically demanding outdoor occupations) may have spent less time outdoors *after* their MeN diagnosis and, therefore, been less likely to contract leptospirosis. We attempted to address this possibility by conducting a sensitivity analysis restricted to cases who had been diagnosed with MeN within the past 3 years, but the results were similar. It is also conceivable that some unknown source of bias operated to depress the risk estimates for both miners/construction workers and the others, such that a true positive association between *Leptospira* seropositivity and MeN for the miners/construction workers looked null and a true null association for the others looked negative. That would be consistent with the hypothesis that it is the interaction of leptospirosis and heat stress (or other occupational hazard) that causes MeN, with both risk factors required.

Other studies that have evaluated the association between *Leptospira* positivity and CKD have reported mixed results. In a study of sugarcane and other workers in the same region, Riefkohl et al.^17^ found some evidence that *Leptospira* seropositivity may be associated with elevated levels of some biomarkers of kidney injury, but their results were inconsistent and, taken together, do not provide strong support for the hypothesis of leptospirosis as a factor in the etiology of MeN. On the other hand, in a study conducted in Taiwan that included a large cross-sectional sample and a smaller prospective cohort, investigators found that, among those with previous exposure to *Leptospira* as determined by the MAT, there was a higher prevalence of CKD, especially of severe CKD, and lower eGFRs.^36^ At the second and last measurement point for the cohort, the average eGFR for people with *Leptospira* titers of ≥ 1:400 was statistically significantly lower than the average eGFR for people with lower titers. Although the kidney biomarker findings were inconsistent with each other, the overall findings are at least suggestive of a relationship between leptospirosis and CKD. In a subsequent article citing this work, one of the investigators postulated two possible mechanisms by which leptospirosis could lead to CKD: 1) progression from leptospirosis-induced AKI and 2) insidious kidney colonization by *Leptospira* followed by a second hit to the kidney from heat stress and/or dehydration.^18^ Our findings do not reinforce these hypotheses.

We found no positive association between hantavirus seropositivity and MeN either, running counter to the results supporting hantavirus as a possible risk factor for CKD in Sri Lanka reported in a brief letter by Gamage et al.^39^ If in reality there is an association, we might have missed it because of contamination/false positives. If in reality there is not an association, Gamage et al. may have found evidence of one because of confounding—their report was too brief to include an explanation of how controls were selected or other steps taken to avoid confounding.

One limitation of our study is the possibility that not all the strains of *Leptospira* circulating in Nicaragua in the last two decades were represented by the set of strains we tested for. *Leptospira noguchii* serovar Nicaragua was one of the “extra” strains we added to CDC’s standard panel of 20. Of our 320 study participants, 57 were positive for this strain; of those 57, 36 (63%) were *negative for all strains in the standard panel*. Thus, it seems crucial to include in testing all the varieties that might have been circulating in Nicaragua in recent years—cross-reactivity among strains cannot be relied on to produce a positive result when only the standard panel of 20 strains is used. Although we included four additional strains, including the reputedly sensitive nonpathogenic *Leptospira biflexa* serovar Patoc, the set may still have been incomplete for Nicaragua, and it is conceivable that some novel, more virulent strain was overlooked.

The major limitation of our study with respect to the infectious disease hypothesis was the impossibility, given our collection of biological specimens at only a single point in time, of determining *whether* infection had in fact occurred (among those with negative serology results) and *when* it had occurred relative to the onset of MeN (among those with positive serology results). Importantly, our case–control design was inadequate for dealing with the phenomenon of waning antibodies. In a serologic study of people infected with *Leptospira* spp. in an outbreak in a nonendemic area of Italy, Lupidi et al.^49^ found that, of the 10 confirmed cases followed for 4.5 years, only one case still had a titer exceeding 1:100 as of that point. Like our main analyses, our post hoc analyses in which cases were restricted to those receiving an MeN diagnosis within the prior 3 years also found no positive association between leptospirosis seropositivity and MeN. These results provide evidence against the more specific hypothesis that infection leads to MeN within a few years. However, questions about whether infection had occurred and the relative timing of infection and MeN remain even when focusing on recently diagnosed MeN cases.

Also, the use of referral by cases and their family members to identify a portion of controls may have caused a bias to the extent that referral was influenced directly by *Leptospira* exposure or, more likely, through an uncontrolled covariate of *Leptospira*. The direction of the bias would be expected to be toward the null because individuals’ social networks generally are more alike in many characteristics than two random people in the population; consequently, cases and controls may be more similar in their exposure distribution than cases and the overall source population.^50^ We think it is more likely that this would have created a stronger bias toward the null for secondary exposures such as occupation and less so for *Leptospira* positivity.

Regarding the self-reported potential risk factors ascertained by means of the survey, we found MeN risk to be associated with mining and construction work, with more than a 4-fold risk of MeN among those who had ever worked in mining or construction compared with those who had not. This finding is in accord with descriptive epidemiology from the same region of Nicaragua^12,13,51,52^ and is consistent with the hypothesis that an occupational hazard such as heat stress is an important factor in the etiology of MeN. The observed inverse relationship between the number of years worked in mining or construction and the risk of MeN we attribute to survival bias.

Although we found no evidence of *Leptospira* or hantavirus seropositivity being risk factors for MeN, the limitations of serology for detecting past infections, especially by *Leptospira*, and our collection of biological specimens at only a single point in time preclude us from definitively ruling out these infections as MeN risk factors on the basis of this study. We consider a prospective cohort design, in which specimens for assessing kidney function and antibody titers are collected at intervals of a few months over the course of several years, to be a more promising approach to studying infectious diseases as potential etiologic agents in MeN.

## Supplementary Files

Supplemental tables
