# Supplementary material for: Investigating Possible Infectious Causes of Chronic Kidney Disease of Unknown Etiology in a Nicaraguan Mining Community
Source: Am J Trop Med Hyg. 2019 Jul 15;101(3):676–83. doi: 10.4269/ajtmh.18-0856 (PMC6726956; doi:10.4269/ajtmh.18-0856)
Supplement: Supplementary file 1 [file tpmd180856.SD1.pdf]

## Supplemental Tables

These tables present the results of the logistic regression models examining the association between *Leptospira* seropositivity and presumptive Mesoamerican nephropathy status. They correspond to the paragraph just above the "Hantavirus serology" heading in the Results section of the paper.

Abbreviations of variables in models

| **Variable name** | **Description** | **Values** |
|---|---|---|
| CLAS2 | Case/control status | 0=control, 1=case |
| AGE | Age in years | |
| AGESQUARED | Age in years squared | |
| SEXCODE | Sex | 0=male, 1=female |
| MM1 | Ever worked in mining or construction | 0=no, 1=yes |
| NOTMM1 | Ever worked in mining or construction | 0=yes, 1=no |
| MMYRS | Number of years worked in mining or construction | |
| LEPTO2 | Seropositive for any tested strain of Leptospira | 0=no, 1=yes |
| MM1*LEPTO2 | Interaction term | |
| NOTMM1*LEPTO2 | Interaction term | |

Set 1.

## Logistic Regression Model CLAS2 = AGE AGESQUARED SEXCODE MM1 MMYRS LEPTO2

| | |
|---|---|
| Number of Observations Read | 288 |
| Number of Observations Used | 288 |

| Response Profile | | |
|---|---|---|
| Ordered Value | CLAS2 | Total Frequency |
| 1 | 0 | 176 |
| 2 | 1 | 112 |

Probability modeled is CLAS2='1'.

| Model Fit Statistics | | |
|---|---|---|
| Criterion | Intercept Only | Intercept and Covariates |
| AIC | 386.911 | 364.993 |
| SC | 390.574 | 390.633 |
| -2 Log L | 384.911 | 350.993 |

| Analysis of Maximum Likelihood Estimates | | | | | |
|---|---|---|---|---|---|
| Parameter | DF | Estimate | Standard Error | Wald Chi-Square | Pr> ChiSq |
| Intercept | 1 | -7.3584 | 1.9615 | 14.0726 | 0.0002 |
| AGE | 1 | 0.2370 | 0.0850 | 7.7790 | 0.0053 |
| AGESQUARED | 1 | -0.00198 | 0.000923 | 4.6079 | 0.0318 |
| SEXCODE | 1 | 0.2063 | 0.5613 | 0.1351 | 0.7132 |
| MM1 | 1 | 1.5721 | 0.4223 | 13.8591 | 0.0002 |
| MMYRS | 1 | -0.0534 | 0.0182 | 8.5732 | 0.0034 |
| LEPTO2 | 1 | -0.5767 | 0.2988 | 3.7247 | 0.0536 |

### Odds Ratio Estimates

| Effect | Point Estimate | Lower 95% Confidence Limit | Upper 95% Confidence Limit |
|---|---|---|---|
| Intercept | .001 | .000 | .030 |
| AGE | 1.267 | 1.073 | 1.497 |
| AGESQUARED | .998 | .996 | 1.000 |
| SEXCODE | 1.229 | .409 | 3.693 |
| MM1 | 4.817 | 2.105 | 11.020 |
| MMYRS | .948 | .915 | .983 |
| LEPTO2 | .562 | .313 | 1.009 |

Set 2.

## Logistic Regression Model CLAS2 = AGE AGESQUARED SEXCODE MM1 MMYRS LEPTO2 MM1*LEPTO2

| | |
|---|---|
| Number of Observations Read | 288 |
| Number of Observations Used | 288 |

| Response Profile | | |
|---|---|---|
| Ordered Value | CLAS2 | Total Frequency |
| 1 | 0 | 176 |
| 2 | 1 | 112 |

Probability modeled is CLAS2='1'.

| Model Fit Statistics | | |
|---|---|---|
| Criterion | Intercept Only | Intercept and Covariates |
| AIC | 386.911 | 361.080 |
| SC | 390.574 | 390.384 |
| -2 Log L | 384.911 | 345.080 |

| Analysis of Maximum Likelihood Estimates | | | | | |
|---|---|---|---|---|---|
| Parameter | DF | Estimate | Standard Error | Wald Chi-Square | Pr> ChiSq |
| Intercept | 1 | -7.0837 | 1.9689 | 12.9440 | 0.0003 |
| AGE | 1 | 0.2401 | 0.0857 | 7.8566 | 0.0051 |
| AGESQUARED | 1 | -0.00201 | 0.000933 | 4.6644 | 0.0308 |
| SEXCODE | 1 | -0.0418 | 0.5785 | 0.0052 | 0.9424 |
| MM1 | 1 | 1.1203 | 0.4564 | 6.0260 | 0.0141 |
| MMYRS | 1 | -0.0520 | 0.0181 | 8.2497 | 0.0041 |
| LEPTO2 | 1 | -2.5111 | 1.0829 | 5.3772 | 0.0204 |
| MM1*LEPTO2 | 1 | 2.2510 | 1.1276 | 3.9852 | 0.0459 |

### Odds Ratio Estimates

| Effect | Point Estimate | Lower 95% Confidence Limit | Upper 95% Confidence Limit |
|---|---|---|---|
| Intercept | .001 | .000 | .040 |
| AGE | 1.271 | 1.075 | 1.504 |
| AGESQUARED | .998 | .996 | 1.000 |
| SEXCODE | .959 | .309 | 2.980 |
| MM1 | 3.066 | 1.253 | 7.499 |
| MMYRS | .949 | .916 | .984 |
| LEPTO2 | .081 | .010 | .678 |
| MM1*LEPTO2 | 9.498 | 1.042 | 86.587 |

Set 3.

## Logistic Regression Model CLAS2 = AGE AGESQUARED SEXCODE NOTMM MMYRS LEPTO2 NOTMM*LEPTO2

| | |
|---|---|
| Number of Observations Read | 288 |
| Number of Observations Used | 288 |

| Response Profile | | |
|---|---|---|
| Ordered Value | CLAS2 | Total Frequency |
| 1 | 0 | 176 |
| 2 | 1 | 112 |

Probability modeled is CLAS2='1'.

| Model Fit Statistics | | |
|---|---|---|
| Criterion | Intercept Only | Intercept and Covariates |
| AIC | 386.911 | 361.080 |
| SC | 390.574 | 390.384 |
| -2 Log L | 384.911 | 345.080 |

| Analysis of Maximum Likelihood Estimates | | | | | |
|---|---|---|---|---|---|
| Parameter | DF | Estimate | Standard Error | Wald Chi-Square | Pr> ChiSq |
| Intercept | 1 | -5.9634 | 1.9069 | 9.7794 | 0.0018 |
| AGE | 1 | 0.2401 | 0.0857 | 7.8566 | 0.0051 |
| AGESQUARED | 1 | -0.00201 | 0.000933 | 4.6644 | 0.0308 |
| SEXCODE | 1 | -0.0418 | 0.5785 | 0.0052 | 0.9424 |
| NOTMM | 1 | -1.1203 | 0.4564 | 6.0260 | 0.0141 |
| MMYRS | 1 | -0.0520 | 0.0181 | 8.2497 | 0.0041 |
| LEPTO2 | 1 | -0.2601 | 0.3262 | 0.6355 | 0.4253 |
| NOTMM*LEPTO2 | 1 | -2.2510 | 1.1276 | 3.9852 | 0.0459 |

### Odds Ratio Estimates

| Effect | Point Estimate | Lower 95% Confidence Limit | Upper 95% Confidence Limit |
|---|---|---|---|
| Intercept | .003 | .000 | .108 |
| AGE | 1.271 | 1.075 | 1.504 |
| AGESQUARED | .998 | .996 | 1.000 |
| SEXCODE | .959 | .309 | 2.980 |
| NOTMM | .326 | .133 | .798 |
| MMYRS | .949 | .916 | .984 |
| LEPTO2 | .771 | .407 | 1.461 |
| NOTMM*LEPTO2 | .105 | .012 | .960 |
